# Senescent endothelial cells are predisposed to SARS-CoV-2 infection and subsequent endothelial dysfunction

**DOI:** 10.1038/s41598-022-15976-z

**Published:** 2022-07-25

**Authors:** Ryota Urata, Koji Ikeda, Ekura Yamazaki, Daisuke Ueno, Akiko Katayama, Masaharu Shin-Ya, Eriko Ohgitani, Osam Mazda, Satoaki Matoba

**Affiliations:** 1 Department of Cardiology, Kyoto Prefectural University of Medicine, 465 Kajii, Kawaramachi-Hirokoji, Kamigyo, Kyoto, 602-8566 Japan; 2 Department of Epidemiology for Longevity and Regional Health, Kyoto Prefectural University of Medicine, 465 Kajii, Kawaramachi-Hirokoji, Kamigyo, Kyoto, 602-8566 Japan; 3 Department of Immunology, Kyoto Prefectural University of Medicine, 465 Kajii, Kawaramachi-Hirokoji, Kamigyo, Kyoto, 602-8566 Japan

**Keywords:** Translational research, SARS-CoV-2

## Abstract

The coronavirus disease 2019 (COVID-19), caused by the novel severe acute respiratory syndrome coronavirus 2 (SARS-CoV-2), remains to spread worldwide. COVID-19 is characterized by the striking high mortality in elderly; however, its mechanistic insights remain unclear. Systemic thrombosis has been highlighted in the pathogenesis of COVID-19, and lung microangiopathy in association with endothelial cells (ECs) injury has been reported by post-mortem analysis of the lungs. Here, we experimentally investigated the SARS-CoV-2 infection in cultured human ECs, and performed a comparative analysis for post-infection molecular events using early passage and replicative senescent ECs. We found that; (1) SARS-CoV-2 infects ECs but does not replicate and disappears in 72 hours without causing severe cell damage, (2) Senescent ECs are highly susceptible to SARS-CoV-2 infection, (3) SARS-CoV-2 infection alters various genes expression, which could cause EC dysfunctions, (4) More genes expression is affected in senescent ECs by SARS-CoV-2 infection than in early passage ECs, which might causes further exacerbated dysfunction in senescent ECs. These data suggest that sustained EC dysfunctions due to SARS-CoV-2 infection may contribute to the microangiopathy in the lungs, leading to deteriorated inflammation and thrombosis in COVID-19. Our data also suggest a possible causative role of EC senescence in the aggravated disease in elder COVID-19 patients.

## Introduction

The pandemic of coronavirus disease 2019 (COVID-19), induced by the novel severe acute respiratory syndrome coronavirus 2 (SARS-CoV-2) infection, has posed a global health emergency. COVID-19 vaccines are certainly effective to prevent infection, severe disease, and hospitalization; however, its spreading remains to be uncontrolled. The striking feature of COVID-19 is the high mortality in elderly; every 1000 people infected with SARS-CoV-2, around 116 will die if they are mid-seventies or older, while little will die if they are under the age of 50^1^. Though elderly is more fragile than younger people in general, this substantial difference in mortality strongly suggest a specific factor(s) that aggravate the disease in elder people.

Post-mortem analysis of the lungs revealed widespread thrombosis with microangiopathy and severe endothelium injury associated with the presence of intracellular virions^2,3^. Furthermore, high incidence of systemic thrombosis such as myocardial infarction and deep vein thrombosis has been reported in COVID-19 patients^4^. Because endothelial cell (EC), which cover the inner surface of entire blood vessels, plays an essential role in the regulation of blood coagulation and fibrinolysis^5,6^, these findings suggest a potential role of EC dysfunction in the pathogenesis of COVID-19. However, it remains controversial whether SARS-CoV-2 infects ECs. There are studies reporting no definite infection of SARS-CoV-2 in ECs using human samples obtained from COVID-19 patients^7,8^ as well. Also, some reports described SARS-CoV-2 infection in cultured human ECs ^9,10^, while other studies showed no SARS-CoV-2 infection in ECs *in vitro*^11,12^.

Cellular senescence has emerged as a fundamental factor for age-related organ dysfunction and diseases. Eliminating senescent cells prevents age-related organ dysfunction in the heart and kidney, and showed beneficial effects in age-related diseases such as osteoarthritis and atherosclerosis^13–15^. Notably, depletion of senescent cells expands the lifespan in mice^16–18^. These findings strongly indicate a central role of cellular senescence in aging and age-related diseases. Furthermore, crucial roles of EC senescence in ageing and age-related diseases have been reported^19–21^. Cellular senescence may therefore contribute to the aggravated disease in elder COVID-19 patients; yet, its causal relevance and underlying mechanisms remain unclear. We here examined SARS-CoV-2 infection in cultured human umbilical vein ECs (HUVECs), and analyzed the post-infection molecular events in early passage and replicative senescent HUVECs, in comparison with influenza A virus.

## Results

### SARS-CoV-2 infects ECs

**Figure 1:**
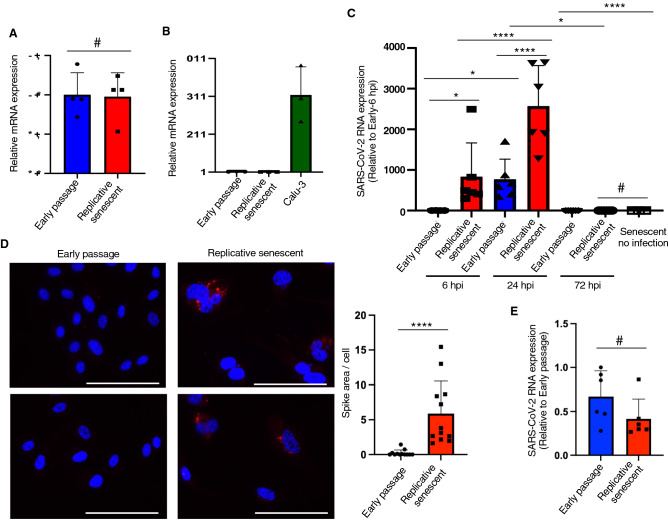
SARS-CoV-2 infects ECs. (**A**) Quantitative PCR analysis for ACE2 in early passage (P3) and replicative senescent (P20) HUVECs (n = 4 each). Target genes expression was normalized to 18S expression levels. (**B**) Quantitative PCR analysis for ACE2 in Calu-3 cells and early passage or replicative senescent ECs (n = 3 each). ACE2 expression was normalized to 18S expression levels. (**C**) Quantitative PCR analysis for SARS-CoV-2 ORF1ab in early passage and replicative senescent ECs infected with SARS-CoV-2 at 1 MOI (n = 4 for senescent EC-no infection; n = 6 each for others). ORF1ab expression was normalized to 18S expression levels. (**D**) Representative images of immunocytochemistry for SARS-CoV-2 spike protein (red fluorescence) in early passage and replicative senescent HUVECs infected with SARS-CoV-2 (50 MOI) at 6 hpi. Bars: 100 μm. Spike-staining was quantitatively analyzed (n = 12 each). (**E**) SARS-CoV-2 attachment on early passage and replicative senescent HUVECs was analyzed using quantitative PCR for ORF1ab (n = 6 each). Statistical analyses were performed using two-tailed unpaired Student’s *t*-test (**A**,**B**), while Mann Whitney *U*-test were used for D and E. One-way ANOVA with Fisher’s LSD post hoc test was used for statistical analysis between multiple groups (**C**). Data are presented as mean ± SE *P < 0.05, ****P < 0.0001 and #Not significant.

SARS-CoV-2 infects the host cell using the angiotensin converting enzyme 2 (ACE2) as a receptor for entry^22^. Therefore, we first examined the expression of ACE2 in young early passage and replicative senescent HUVECs. Cellular senescence was confirmed by enhanced p21 expression assessed by immunoblotting, and senescence-associated SPiDER-β-Gal staining (Supplementary Fig. 1). ACE2 mRNA expression levels were similar between young and senescent ECs assessed by quantitative PCR (Fig. 1A). However, its expression levels in ECs were remarkably low when compared to that in Calu-3 cells, a cell line of lung epithelial cells that are often used for SARS-CoV-2 infection experiments (Fig. 1B). Moreover, we failed to detect ACE2 protein expression in these HUVECs using immunoblotting and immunocytochemistry. Considering the high Ct-value in HUVECs (33.15 ± 0.37 for early passage ECs; 33.64 ± 0.33 for replicative senescent ECs vs. 22.10 ± 0.48 for Calu-3 cells), HUVECs express very little or no ACE2, which is consistent with the previous reports^10^.

To investigate the SARS-CoV-2 infection in ECs, we incubated these HUVECs with SARS-CoV-2 at 1 MOI for 1 h, followed by incubation with fresh growth medium. RNAs were collected from the cells, and the SARS-CoV-2 infection was assessed by analyzing virus RNA (open reading frame1 ab; ORF1ab) levels using quantitative RT-PCR^23,24^. SARS-CoV-2 RNA was detected in cellular RNAs, indicating the virus entry into ECs (Fig. 1C). Of note, SARS-CoV-2 RNA levels were significantly higher in senescent HUVECs than in early passage cells, especially at early time point after infection (Fig. 1C). However, SARS-CoV-2 RNA levels in the cells substantially reduced and became indetectable at 72 h post infection (hpi) (Fig. 1C). We further assessed virus infection by immunostaining for SARS-CoV-2 spike protein. Consistent with the virus RNA qPCR analysis, significant SARS-CoV-2 spike staining was observed in senescent HUVECs, while the spike staining was barely detectable in early passage HUVECs (Fig. 1D).

Because of the remarkable difference of virus RNA levels at early time of infection, we performed a virus attachment assay. SARS-CoV-2 attachment was not different between early passage and senescent HUVECs (Fig. 1E). Therefore, virus entry after attachment should be enhanced in replicative senescent HUVECs comparing to that in early passage cells. These data collectively suggest that SARS-CoV-2 indeed infects ECs, and EC cellular senescence enhances virus infection, while SARS-CoV-2 seems not to replicate in ECs and disappears from the cells soon.

### SARS-CoV-2 enters into ECs through endocytosis

**Figure 2:**
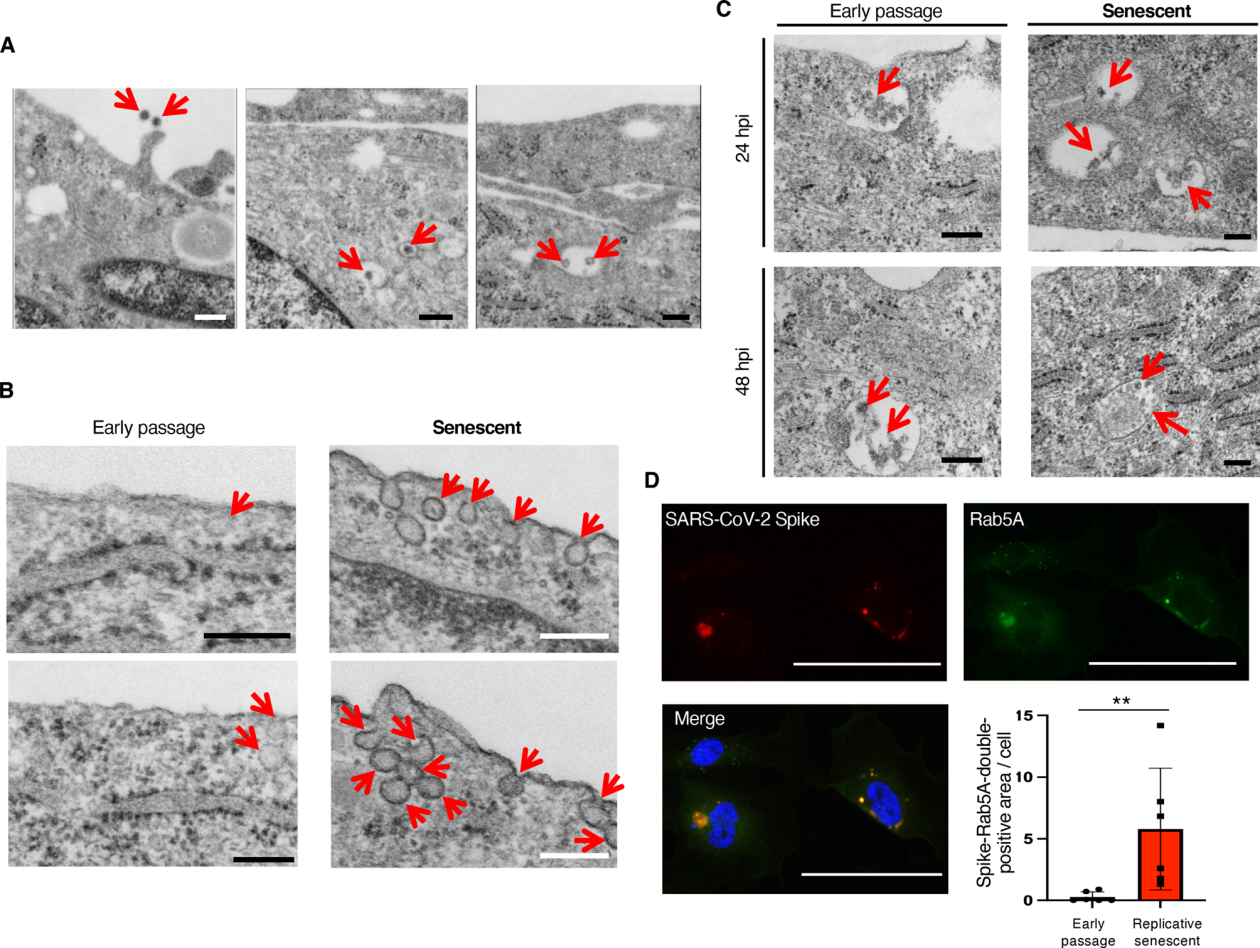
SARS-CoV-2 enters into ECs through endocytosis. (**A**) Representative electron microscopy images (× 80,000) for senescent HUVECs infected with SARS-CoV-2 at 50 MOI (2 hpi). Arrows indicate SARS-CoV-2 virions. Bars: 200 nm. (**B**) Representative electron microscopy images (× 80,000) for early passage and senescent ECs infected with SARS-CoV-2 at 1 MOI (6 hpi). Arrows indicate SARS-CoV-2 entry via caveolae-like structures. Bars: 200 nm. (**C**) Representative electron microscopy images (× 80,000) for early passage and senescent ECs infected with SARS-CoV-2 at 1 MOI (24 and 48 hpi). Arrows indicate SARS-CoV-2 virions. Bars: 200 nm. (**D**) Representative images of immunocytochemistry for SARS-CoV-2 spike protein (red fluorescence) and Rab5A (green fluorescence) in replicative senescent HUVECs infected with SARS-CoV-2 (50 MOI) at 6 hpi. SARS-CoV-2 spike proteins were largely co-localized with Rab5A. Bars: 100 μm. Spike and Rab5A double-positive areas were quantitatively analyzed (n = 6 each). Statistical analysis was performed using Mann Whitney *U*-test. Data are presented as mean ± SE **P < 0.01.

We further confirmed SARS-CoV-2 infection by detecting intracellular virions using transmission electron microscopy. Intracellular virions surrounded by endosome-like structure were detected in senescent HUVECs infected with SARS-CoV-2 at 2 hpi (Fig. 2A). At 6 hpi, many SARS-CoV-2 entry via caveolae-like structures was observed in senescent HUVECs, while much less virus entry was detected in early passage HUVECs (Fig. 2B). These observations are consistent with the remarkable increase of virus RNA in senescent HUVECs at early time point after infection. Observation at later time point revealed that virions accumulated in endosome-like structures at 24 and 48 hpi, and they appeared to be disrupted at 48 hpi (Fig. 2C). We then performed double staining for SARS-CoV-2 spike protein and Rab5A, an endosome marker protein, and found that virus spike proteins were largely localized in endosomes (Fig. 2D). These data collectively suggest that SARS-CoV-2 enters into ECs through endocytosis, at least partially via caveolae, but is unable to export their RNAs from the endosome-like structure, and therefore cannot replicate in ECs.

Notably, both early passage and senescent HUVECs infected with SARS-CoV-2 appeared normal, and no significant cell death and morphological changes were detected at 72 hpi (Supplementary Fig. 2). These data suggest that SARS-CoV-2 infection unlikely causes severe injury in ECs, and therefore, the severe endothelial injury detected in post-mortem lung analysis of COVID-19 may have been induced by excessive cytokines released by immune cells, but not by virus infection itself.

We then analyzed the influenza A (INFA) infection in ECs to compare with the SARS-CoV-2 infection. Quantitative analysis of INFA M gene RNA^25^ in HUVECs infected with INFA showed the highest levels at 24 hpi, and substantial reduction was observed at 72 hpi (Supplementary Fig. 3). Also, INFA RNA levels were higher in senescent HUVECs than in early passage cells at 24 hpi. These INFA RNA expression patterns after infection are similar to that of SARS-CoV-2 infection; however, the enhanced infection rate in senescent HUVECs at early time point after infection (6 hpi) was observed only for SARS-CoV-2.

### SARS-CoV-2 infection affects various genes expressions in ECs

**Figure 3:**
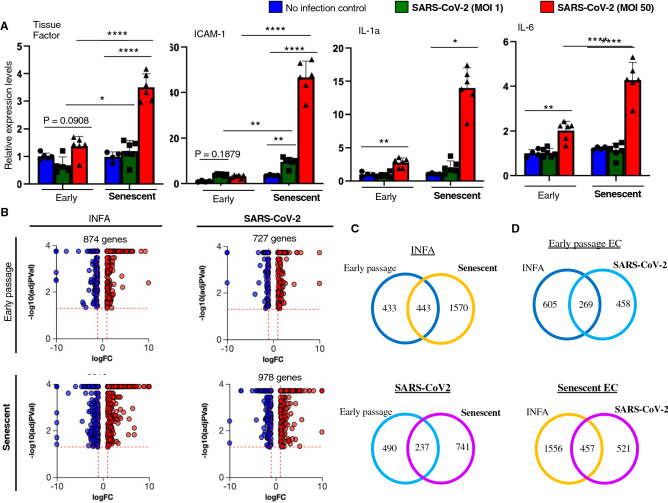
SARS-CoV-2 infection affects various genes expression in ECs. (**A**) Quantitative PCR analysis for tissue factor (TF), intercellular adhesion molecule-1 (ICAM-1), interleukin-1a (IL-1a), and IL-6 in early passage and senescent HUVECs with or without SARS-CoV-2 infection (1 or 50 MOI) at 72 hpi (n = 4 each for control cells; n = 6 each for infected cells). Statistical analyses were performed using one-way ANOVA with Fisher’s LSD post hoc analysis for TF, ICAM-1, and IL-6, while Kruskal-Wallis test by ranks was used for IL-1a. Data are presented as mean ± SE *P < 0.05, **P < 0.01, and ****P < 0.0001. (**B**) Comprehensive RNA-Seq data analysis in ECs infected with SARS-CoV-2 or INFA was performed. Volcano plots for genes whose expression was significantly altered (*P *< 0.05) by SARS-CoV-2 or INFA infection in early passage and senescent ECs were shown (n = 3 each). Red plots indicate the genes significantly increased more than 2-fold after the virus infection. Blue plots indicate the genes significantly (*P *< 0.05) decreased less than half after the virus infection. (**C**,**D**) Venn Diagram analysis of the genes whose expression was significantly altered by SARS-CoV-2 or INFA infection (> 2 or < 1/2 fold) in young and senescent ECs. Numbers indicate the number of genes included in the sets of the intersections.

We analyzed molecular events occurred in ECs after SARS-CoV-2 infection. Interestingly, genes involved in blood coagulation, leukocyte recruitment, and inflammation were upregulated in ECs after SARS-CoV-2 infection (Fig. 3A). Notably, these transcriptional changes were markedly enhanced in senescent HUVECs compared to those in early passage HUVECs (Fig. 3A). Because tissue factor (TF) is a potent inducer for blood coagulation, we further analyzed TF protein expression using immunocytochemistry. Significant TF-staining was observed in senescent HUVECs after SARS-CoV-2 infection, while TF-staining was very faint or undetectable in infected early passage cells (Supplementary Fig. 4). These data suggest that SARS-CoV-2 infection in senescent ECs might cause enhanced blood coagulation through the high TF expression. Also, we assessed proliferation capacity in infected ECs, and found no effect of SARS-CoV-2 infection on proliferation capacity in both early passage and senescent HUVECs (Supplementary Fig. 5).

We then performed comprehensive mRNA expression analyses by RNA-Seq. RNAs expression was analyzed in early passage and senescent HUVECs before and after SARS-CoV-2 or INFA infection at 1 MOI (at 72 hpi), and the alteration in gene expression was explored. Many genes expression was altered by virus infection, although the virus RNAs were mostly indetectable at this time point (Fig. 3B). Of note, the number of genes of which expression was significantly altered by virus infection was greater in senescent HUVECs than in early passage HUVECs in both cases of SARS-CoV-2 and INFA infection (Fig. 3B).

Gene ontology (GO) analysis for SARS-CoV-2 infection-related genes revealed 630 enriched GOs in early passage HUVECs, and 1047 enriched GOs in senescent HUVECs. When classified top 100 GOs by functions, different trend was observed in early passage and senescent HUVECs. In early passage HUVECs, more GOs related with cell cycle/proliferation and DNA process were detected, while GOs related with responses to stress and viruses were more prevalent in senescent HUVECs (Supplementary Fig. 6). Similar number of GOs related with inflammation was detected in early passage and senescent HUVECs.

Venn diagram analysis showed that less than 50% of genes were commonly affected by each virus infection when compare between early passage and senescent HUVECs (Fig. 3C). Furthermore, genes commonly affected by SARS-CoV-2 and INFA infection were minor, and many genes were affected uniquely by SARS-CoV-2 and INFA infection in ECs (Fig. 3D). These data suggest that molecular events induced by virus infection in ECs varies depending on the types of viruses, and that cellular responses to the virus infection differ between early passage and senescent HUVECs.

**Figure 4:**
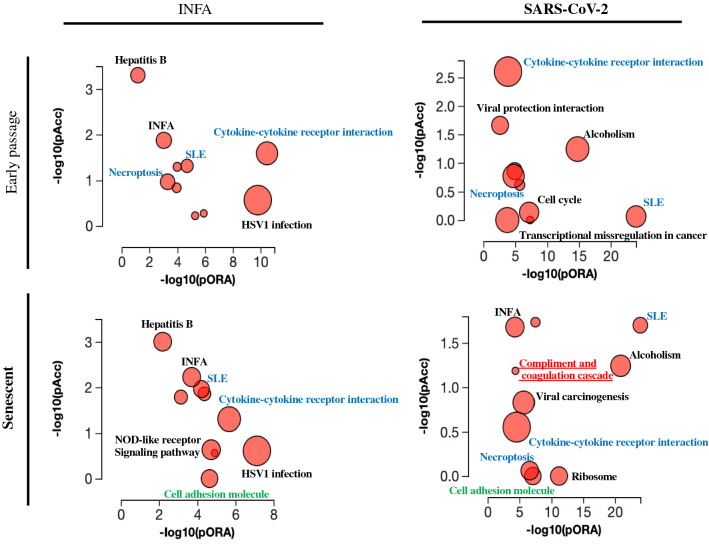
Pathway analysis for the genes whose expression was significantly altered by the virus infection. Pathways affected by the virus infection were analyzed using iPathwayGuide. Dots representing the top 10 impacted pathways are positioned by their p-values from two different analyses; an Impact analysis measuring total perturbation accumulation (pAcc) and an Over-representation analysis (pORA). The size of each dot denotes the total number of genes in the corresponding pathway.

Pathway analysis for genes whose expression was significantly altered by twofold after the virus infection showed that various pathways were affected by the virus infection (Fig. 4). Pathways for inflammation and immune response were commonly affected, while the pathway for coagulation cascades showed up only in senescent HUVECs infected with SARS-CoV-2 (Fig. 4). These data strongly suggest a significant impact of SARS-CoV-2 infection in EC functions, and a possible contribution of the virus infection in ECs in the incidence of COVID-19-associated thrombosis, especially in elder patients.

## Discussion

After the first report, COVID-19 has been immediately spread worldwide, and its pandemic is still uncontrolled. Striking feature of COVID-19 is the high mortality in elderly and the high incidence of systemic thromboembolisms. Because of the critical role of ECs in thrombosis, we explored the effects of SARS-CoV-2 infection in ECs using young early passage and replicative senescent cultured human ECs. Although post-mortem histological analysis of the lungs of COVID-19 patients showed severe endotheliitis, it was not conclusive whether SARS-CoV-2 indeed infects and injures ECs^8,26^. Furthermore, effects of SARS-CoV-2 infection on EC functions remained unknown, while a direct effect of SARS-CoV-2 spike protein in EC functions via downregulation of ACE2 has been reported^27^. In this study, we identify that senescent ECs are predisposed to SARS-CoV-2 infection, and infection-related dysfunction was augmented in senescent ECs as compared to early passage cells. When compare the Ct-value for SARS-CoV-2 ORF1ab RNA (~32) to that for INFA M gene RNA (~18) in early passage HUVECs 24 h after infection, it appears that SARS-CoV-2 infectivity into ECs is quite low. This is consistent with the very low expression levels of ACE2 in ECs comparing to those in Calu-3 cells. Because several reports described ACE2-independent infection of SARS-CoV-2^28–30^, SARS-CoV-2 may infect in HUVECs in an ACE2-independent manner, especially when ECs become senescent. Furthermore, our data showed that SARS-CoV-2 RNA disappears from the cells 72 h after infection. These data are consistent with the previous report showing temporary SARS-CoV-2 infection in cultured human coronary artery ECs^10^. In this previous study, SARS-CoV-2 infection was not detected in HUVECs assessed by immunostaining for spike protein. In our study, spike staining was barely detectable in early passage HUVECs either, while they were readily detectable in senescent HUVECs. Although the virus spike protein was not detected by immunostaining, virus RNA was detected in early passage HUVECs by quantitative PCR. Furthermore, many genes expression was altered in early passage HUVECs after exposure to SARS-CoV-2; therefore, we think SARS-CoV-2 indeed infects early passage HUVECs, though its infectivity seems to be quite low. These low infectivity and transient infection may cause the difficulties to detect SARS-CoV-2 viruses in endothelium of human samples obtained from COVID-19 patients, although SARS-CoV-2 has an ability to infect ECs.

Our electron microscopic and immunocytochemistry analyses suggest that SARS-CoV-2 may enter into ECs through endocytosis. The best characterized endocytic routes are clathrin- and caveolae-mediated endocytosis^31^. Many viruses use these endocytic pathways to enter host cells^32–34^. It has been reported that SARS-CoV-2 enters the cells through clathrin-mediated endocytosis^35^, while contribution of caveolae-mediated endocytosis for SARS-CoV-2 infection remains unclear. Our electron microscopic data suggest that SARS-CoV-2 enters into ECs through caveolae-mediated endocytosis, and this pathway is remarkably enhanced in senescent ECs comparing to early passage cells. It has been reported that caveolin-1, the most important structural protein of caveolae, is upregulated in senescent cells^31^. Given that virus attachment was not different between early passage and senescent HUVECs, enhanced infectivity of SARS-CoV-2 in senescent ECs might be attributed to the senescence-associated increase of caveolae-mediated endocytosis; yet, further analysis is certainly required to elucidate its underlying mechanisms.

Our data showed that SARS-CoV-2 remains and accumulates in endosomes even 48 hours after infection, and they disappear from the cells in 72 h after infection. These data suggest that SARS-CoV-2 is not able to export the genomic RNA into the cytosol, and therefore is unable to replicates in ECs. Accordingly, SARS-CoV-2 infection seems not to injure ECs as it dose in airway epithelial cells. Nevertheless, SARS-CoV-2 infection affected wide variety of genes expression in ECs. Cells sense the microbial infection largely through pattern recognition receptors such as Toll-like receptors (TLR), and some of TLRs are expressed at endosome to detect microorganisms entered through endocytosis^36^. Also, it has been reported that SARS-CoV-2 induces pro-inflammatory responses in macrophages via TLR-4^37^. Therefore, virus retention in endosome could lead to persistent activation of TLR-downstream signaling pathway such as NF-κB, resulting in the alteration of various genes expression. Further analyses are required to elucidate the signaling pathways through which SARS-CoV-2 infection alters gene expressions in ECs.

In this study, we used Wuhan strain of SARS-CoV-2 to analyze its infectivity in human cultured ECs. However, many variants that show different clinical features emerged, and the Wuhan strain is no longer a major strain for COVID-19; therefore, analysis for infectivity of SARS-CoV-2 variants in senescent ECs is needed, and the analysis may provide mechanistic insights into variant-dependent unique clinical features. We used PR8 influenza A that is a mouse-adapted strain to explore the influenza infection in ECs in this study. It is notable that the findings using this strain cannot be generalized to other strains of influenza. Also, we used HUVECs to assess the virus infection in ECs in this study. Because the lungs are the major target tissue for SARS-CoV-2 infection, using ECs derived from lung microvessels, and compare the results with current study using HUVECs will provide additional important information.

In the current study, we revealed that cellular senescence enhances SARS-CoV-2 infection in ECs, and accentuates post infection molecular events including the modification of the pathway for coagulation. It has been reported that senolytics, which selectively kill senescent cells, reduced coronavirus-related mortality in old mice^38^, which support a crucial role of senescent ECs in the pathology of COVID-19. Analyses using young and aged mice infected with SARS-CoV-2 is a strong approach to observe infection-related microthrombi or barrier dysfunction in association with aging and/or cellular senescence, which should be done in the future. The sustained EC dysfunctions caused by SARS-CoV-2 infection may contribute to the microangiopathy in the lungs and to the prevalence of thromboembolisms in COVID-19, especially in elder patients in association with EC senescence.

## Materials and methods

### Materials

SARS-CoV-2 (JPN/TY/WK-521) was obtained from the National Institute of Infectious Diseases, Japan. Influenza A (A/Puerto Rico/8/34 (H1N1), PR8) was kindly provided by Dr. Hideki Hasegawa (National Institute of Infectious Diseases). Human umbilical vein endothelial cells (HUVECs) were obtained from LIFELINE Cell Technology (#FC-0003). Anti-Spike antibody (SA39) was generated by Cell Engineering Corporation (Osaka, JAPAN). Antibodies for p21 (#2947), γH2AX (#9718), Rab5a (#46449), and Tissue factor (#55147) were obtained from Cell Signaling Technology. Antibody for GAPDH (#ab105428) was obtained from Abcam. Antibody for Ki-67 (#M7240) was obtained from Dako. Alexa594-anti-rabbit IgG (#A21207), Alexa488-anti-mouse IgG (#A11029), Alexa594-anti-mouse IgG (#A11005) were obtained from Invitrogen.

### Cell culture

Cells were regularly cultured in the CO_2_ incubator for cells at 37 °C under 5% CO_2_ levels. Replicative senescent ECs were prepared as previously described^19^. Briefly, HUVECs were cultured in HuMedia-EG2 medium (Kurabo #KE-2150S), and regularly passaged at 1:4 ratio when reached sub-confluent. Cells were passaged until they did not show obvious proliferation with enlarged and flattened morphology (usually passage 18–20). Cellular senescence was confirmed by enhanced senescence-associated β-GAL activity; reduced proliferation; increased expression of senescence-associated genes such as p16 and p21; accumulated DNA damage assessed by immunocytochemistry for γH2AX^19^. P3 HUVECs were regularly used as young early passage ECs. For virus infection, 1 × 10^5^ cells were plated on 12-well plate, and incubated with SARS-CoV-2 (at MOI 1 or 50) or influenza A (at MOI 1) for 1 h with gentle rotation, followed by changing medium with fresh growth one. RNAs were extracted from the cells 6, 24, and 72 h after the infection.

### Attachment assay

SARS-CoV-2 attachment assay was performed as previously reported^39,40^. Briefly, early passage and replicative senescent HUVECs were detached from culture plates using versene solution, followed by washing with binding buffer (DMEM containing 2% FBS). Cells (2 × 10^5^) were resuspended in 100 μl binding buffer, and then mixed with SARS-CoV-2 at 50 MOI. After incubation at 4 °C for 2 h, cells were washed twice with binding buffer to eliminate unbound viruses, followed by lysis in TRIzol (Invitrogen #15596026). Virus attachment was quantitatively analyzed using real-time PCR.

### Quantitative PCR

RNAs were isolated from the cells using TRIzol, and then purified using NucleoSpin RNA clean-up kit (Macherey-Nagel #U0955). Subsequently, cDNA was synthesized using PrimeScript RT Master Mix (TaKaRa #RR036), followed by quantitative PCR using CFX384 (BioRad). Primers used were shown in Supplementary Table 1. The target gene mRNA and virus RNA expression levels were normalized to 18S expression levels. The relative gene expressions are presented in arbitrary units. RNAs were also subjected to RNA-Seq analyses. Library was prepared using TruSeq RNA Exome (Illumina #20020189), and paired-end RNA-Seq was performed using NovaSeq600 (Illumina). The data was analyzed using the iPathwayGuide (ADVAITA). The csCluster command of cummerbund was used to perform K-means clustering. Genes whose expression changes by more than 1.5-fold were included for analysis. Gene Ontology (GO) term analysis of gene clusters was performed using the goseq.

### Immunoblotting

Cells were lysed in RIPA buffer containing protease inhibitor cocktail (Sigma #P8340). After measurements using DC protein assay kit (BioRad #500112), the same amount of proteins were run on SDS-PAGE gel, followed by transfer onto nitrocellulose membrane. The membrane was incubated with p21 (1:1000), or GAPDH (1:3000) antibody at 4 °C for overnight, following blocking with 10% skim milk. After washing with TBS-T, the membrane was incubated with HRP-labelled secondary antibody (1:2000), followed by detection using ChemiDoc Touch MP (BioRad).

### Immunocytochemistry

Cells were fixed using 4% PFA, followed by permeabilization with 0.1% Triton-X. After blocking with 10% normal donkey serum, cells were incubated with Spike (1:500), Rab5A (1:400), Ki-67 (1:200), γH2AX (1:200), or TF (1:100) antibody at 4 °C for overnight. After washing with PBS, cells were incubated with fluorescence-labelled secondary antibody (1:500). Subsequently, cells were mounted with antifade mounting medium with DAPI (Vector Laboratory #H-1200), followed by analysis under all-in-one fluorescence microscopy (Keyence). Spike staining-positive areas were quantified using the Image-J software, and presented in arbitrary units.

### SPiDER-β-Gal staining

Early passage and replicative senescent ECs plated on 48-well plate were washed with HBSS, followed by incubation with 50 nM Bafilomycin A1 in 5% CO_2_ incubator for 1 h. Cells were then incubated with SPiDER-β-Gal solution (Dojindo #SG-2; 1:1000) and 10 μg/ml Hoechst 33342 in 5% CO_2_ incubator for 30 min. After washing with HBSS, cells were observed under all-in-one fluorescence microscopy (Keyence).

### Transmission electron microscopy

Cells were fixed with 2.5% glutaraldehyde at room temperature for 30 min, followed by post-fixation with 2% OsO4 at 4 °C for 2 h. After dehydration, samples were infiltrated with propylene oxide, followed by embedding with Quetol812. Sections were prepared using ultramicrotome, stained with uranyl acetate, and then analyzed under electron microscopy (Hitachi H-7500).

### Statistical analysis

All data are presented as mean ± S.E. Data normality was assessed using Shapiro-Wilk test. The difference between 2 groups was analyzed using two-tailed unpaired Student’s *t*-test, while differences between groups more than 3 were analyzed using one-way ANOVA with Fisher’s LSD post hoc test. In case the data normality was not shown, the differences between groups were analyzed using non parametric Mann-Whiney *U*-test for comparison between 2 groups or Kruskal-Wallis test by ranks for comparison between groups more than 3. *P *< 0.05 was considered statistically significant.

## Supplementary Information

Supplementary Information 1.

Supplementary Information 2.

## Data Availability

The authors declare that all data supporting the findings of this study are available within the paper. The datasets generated and/or analyzed during the current study are available in the Gene Expression Omnibus (GEO) repository (GES206677).
