# Supplementary material for: Senescent endothelial cells are predisposed to SARS-CoV-2 infection and subsequent endothelial dysfunction
Source: Sci Rep. 2022 Jul 25;12:11855. doi: 10.1038/s41598-022-15976-z (PMC9314328; doi:10.1038/s41598-022-15976-z)
Supplement: Supplementary file 1 — Supplementary Information 1. [file 41598_2022_15976_MOESM1_ESM.pdf]

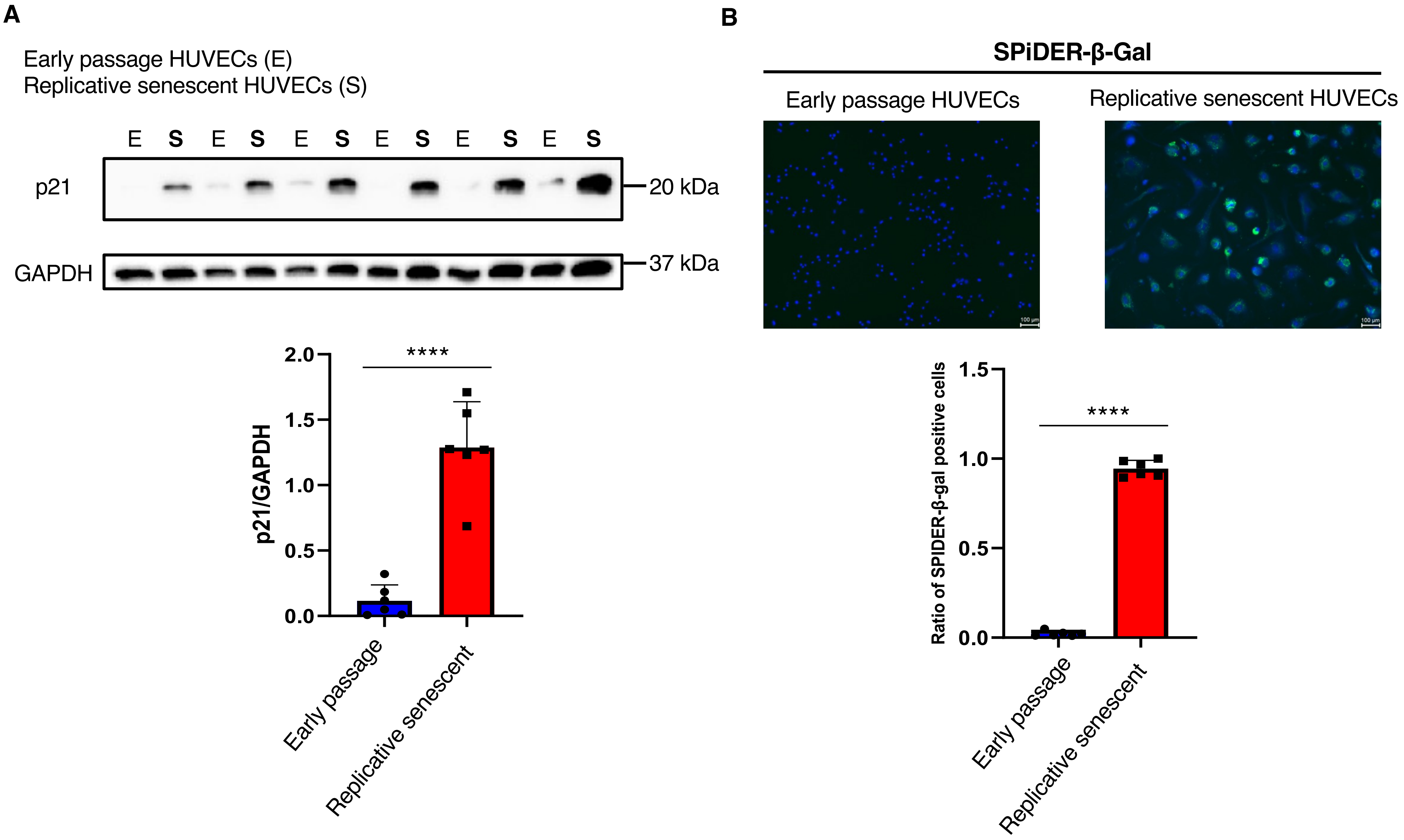

Supplementary Figure-1.
(**A**) Immunoblotting for p21 and GAPDH in early passage and replicative senescent HUVECs. Uncropped blots were shown in Supplementary Fig. 7. (**B**) SPiDER-β-Gal staining (green fluorescence) in early passage and replicative senescent HUVECs. The difference between the groups was analyzed by two-tailed unpaired Student's *t*-test. Data are presented as mean ± S.E. ****$P < 0.0001$.

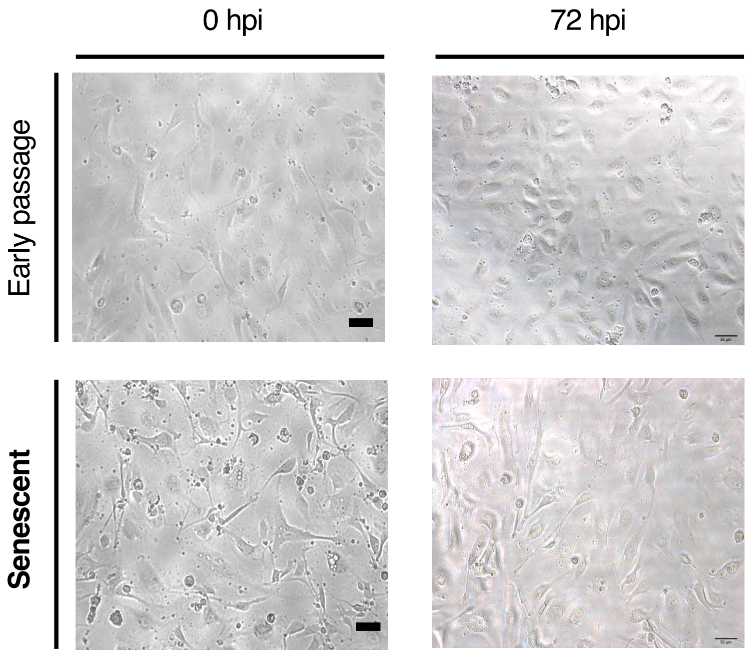

Supplementary Figure-2.
Representative phase contrast microscopy images for early passage and replicative senescent HUVECs infected with SARS-CoV-2 at 50 MOI (0 and 72 hpi). Bars: 50 μm.

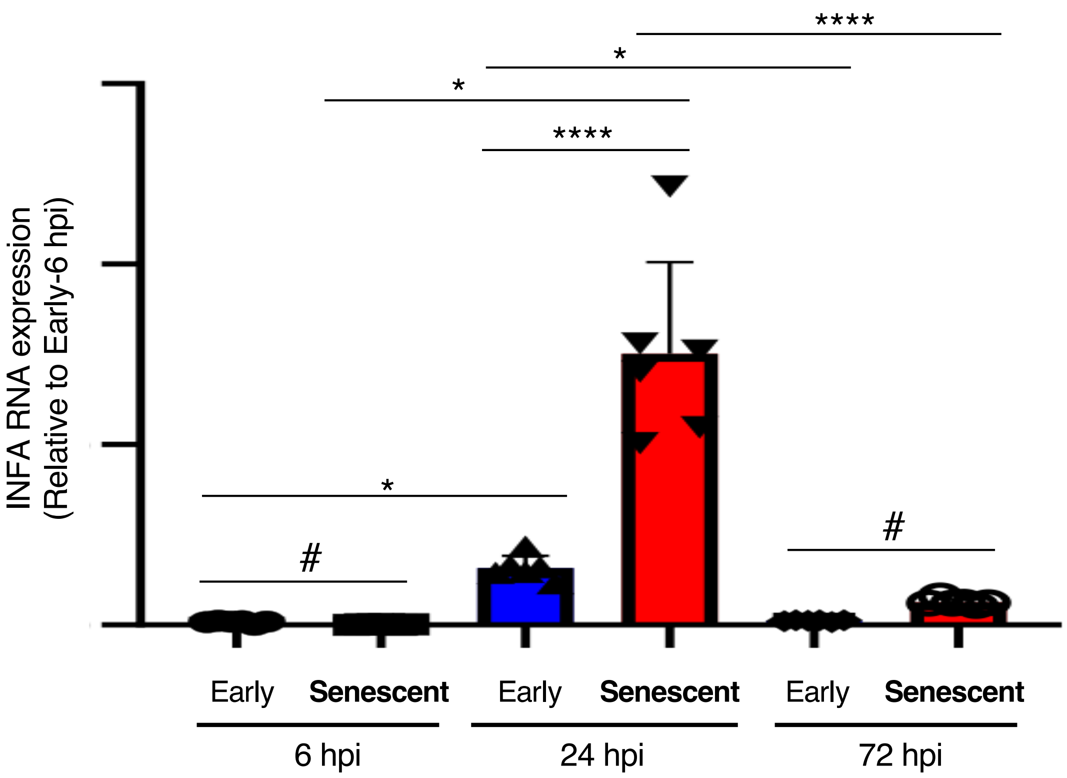

Supplementary Figure-3.
Quantitative PCR analysis for INFA M gene in early passage and replicative senescent HUVECs infected with INFA at 1 MOI (n = 5 for young-6 h; n = 6 each for others). M gene expression was normalized to 18S expression levels. One-way ANOVA with Fisher's LSD post hoc test was used for difference evaluation between the groups. Data are presented as mean ± S.E. $^{*}P < 0.05$, $^{****}P < 0.0001$ and #Not significant.

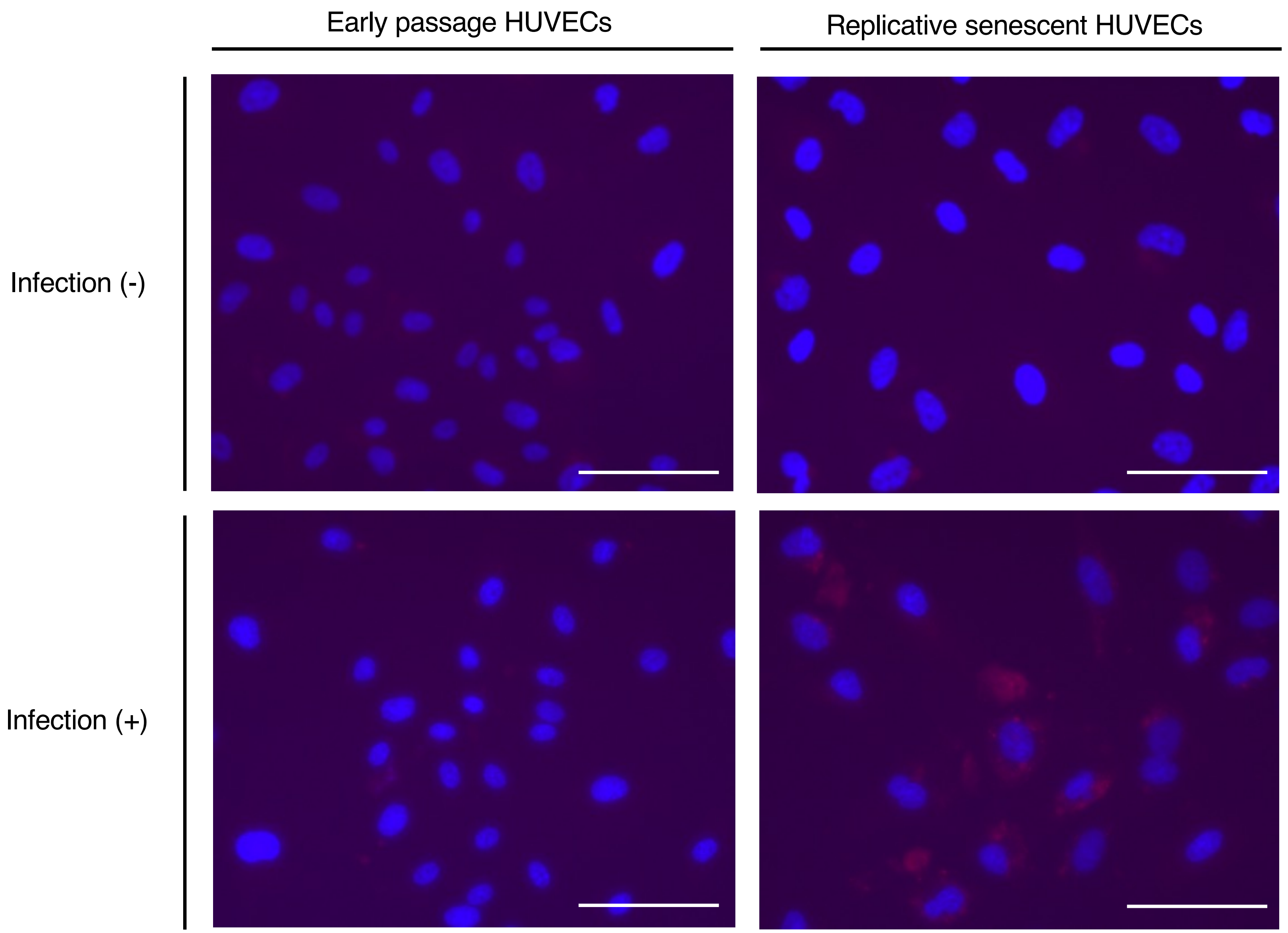

Supplementary Figure-4.
Representative images of immunocytochemistry for tissue factor (red fluorescence) in early passage and replicative senescent HUVECs with or without SARS-CoV-2 infection at 50 MOI (at 48 hpi). Bars: 100 μm.

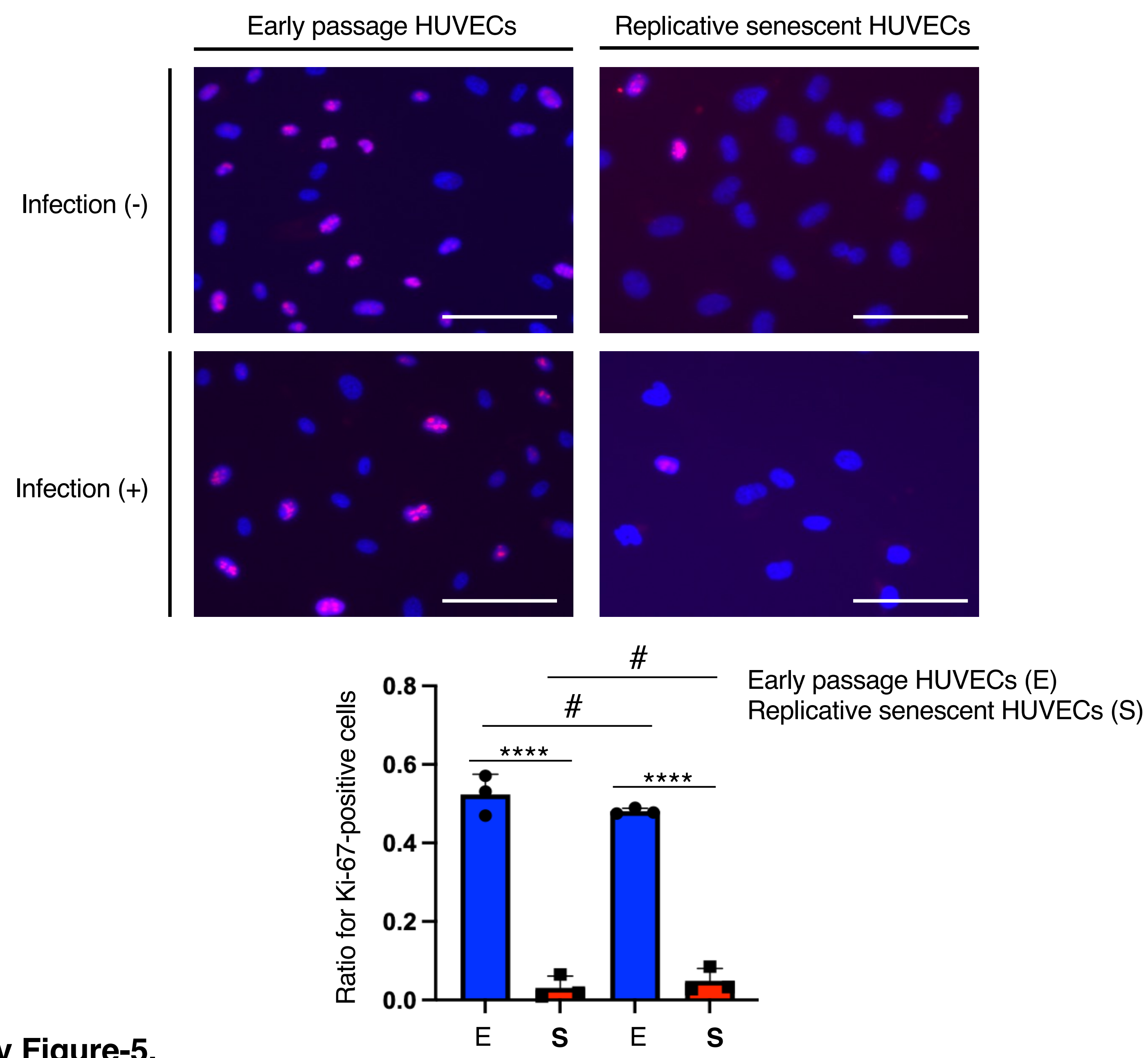

Supplementary Figure-5.
Representative images of immunocytochemistry for Ki-67 (red fluorescence) in early passage and replicative senescent HUVECs with or without SARS-CoV-2 infection at 50 MOI (at 48 hpi). Bars: 100 μm. One-way ANOVA with Fisher's LSD post hoc test was used for difference evaluation between the groups. Data are presented as mean ± S.E. ****$P < 0.0001$ and #Not significant.

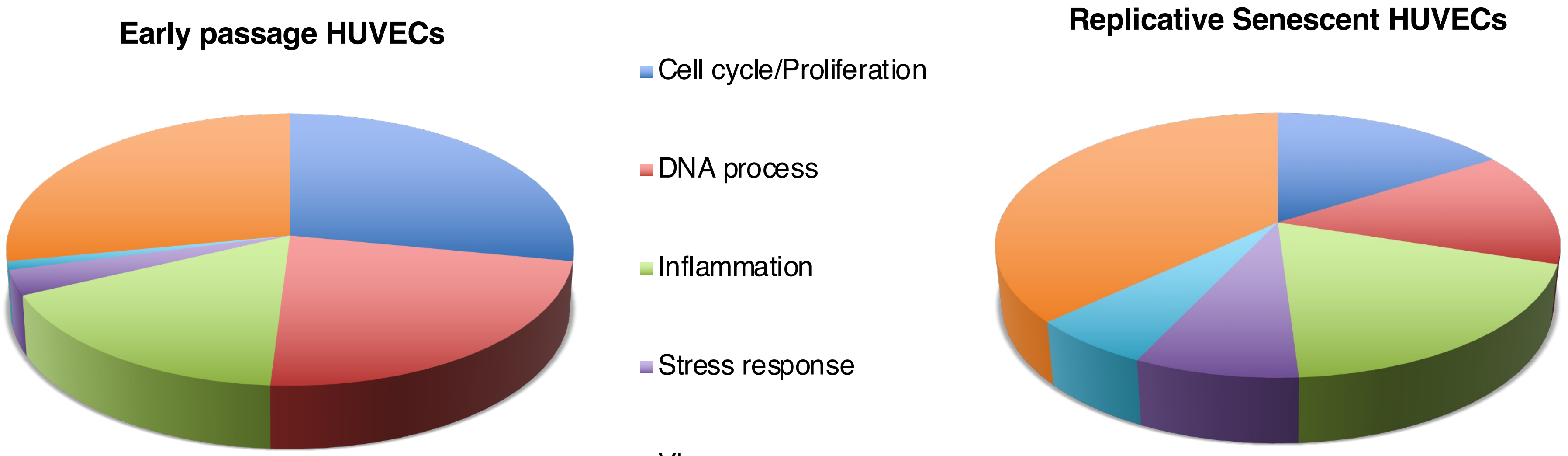

Supplementary Figure-6.
Classification of top 100 enriched GOs in SARS-CoV-2-infecteion-related genes in early passage and replicative senescent HUVECs (n = 3 each).

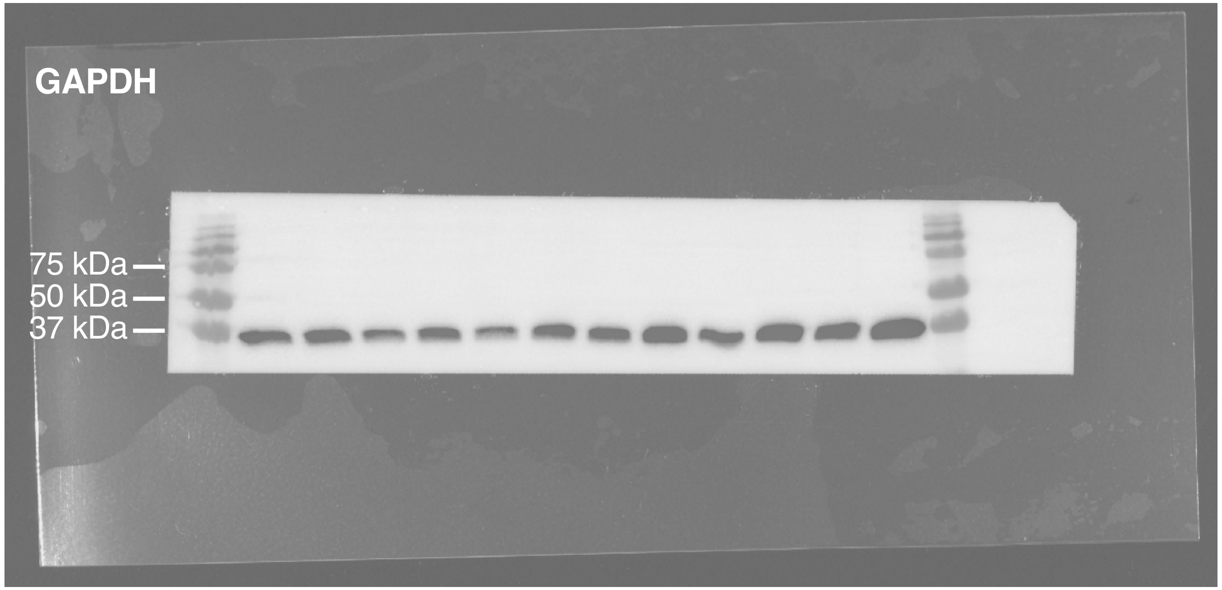

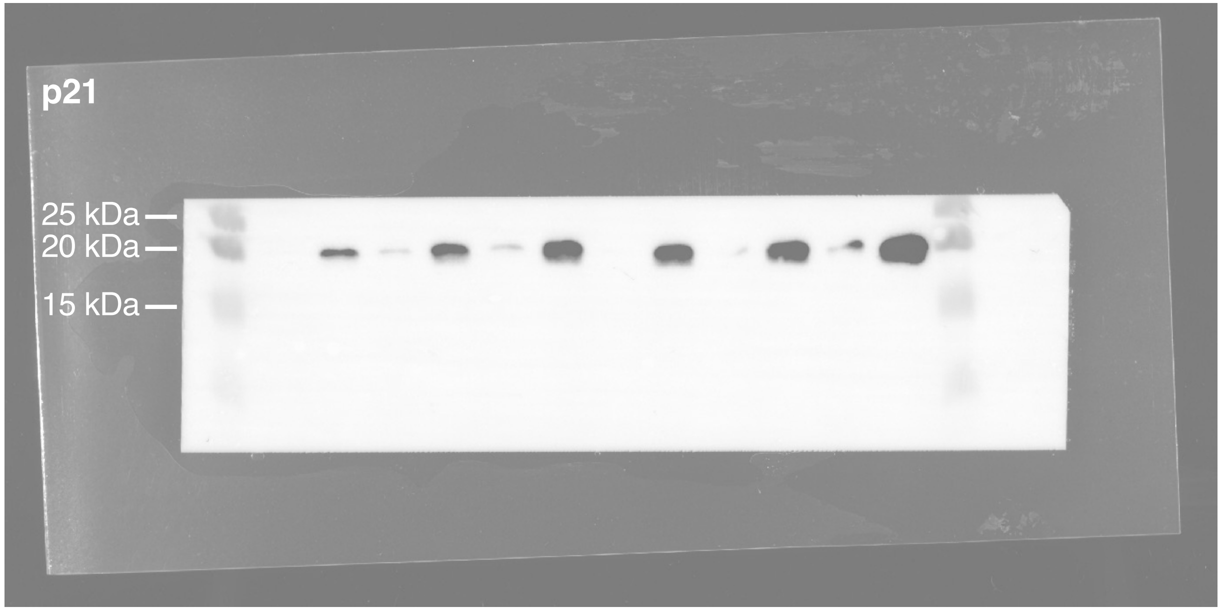

Supplementary Figure-7.
Uncropped blots shown in Supplementary Fig. 1. The membrane was cut into 2 pieces before incubation with the 1st antibodies. The upper membrane was used for the GAPDH, while the lower one was used for the p21 immunoblotting.
