# Supplementary material for: Senescent endothelial cells are predisposed to SARS-CoV-2 infection and subsequent endothelial dysfunction
Source: Sci Rep. 2022 Jul 25;12:11855. doi: 10.1038/s41598-022-15976-z (PMC9314328; doi:10.1038/s41598-022-15976-z)
Supplement: Supplementary file 2 — Supplementary Information 2. [file 41598_2022_15976_MOESM2_ESM.docx]

**Supplementary Table-1**

**Nucleotide sequence of primers**

| SARS-CoV-2 ORF1ab | CCCTGTGGGTTTTACACTTAA |
| --- | --- |
|  | ACGATTGTGCATCAGCTGA |
| INFA M gene | GGACTGCAGCGTAGACGCTT |
|  | CATCCTGTTGTATATGAGGCCCAT |
| 18S | GTAACCCGTTGAACCCCATT |
|  | CCATCCAATCGGTAGTAGCG |
| IL1α | GGTCACCAAATTCTACTTCCAGGAGGAC |
|  | GTGACCAGGTTGTTGTGACGCCTTC |
| IL6 | GAAGCTGCAGGCACAGAACCAGTGGC |
|  | CTGACCAGAAGAAGGAATGCCCATT |
| ACE2 | GAATGTAAGGCCACTGCTCAACTAC |
|  | TTTGGTCTGCATATGGACTCCAGTCG |
| TF | GGAGCATGAAGACCCTGGAGTTCAAA |
|  | CTTGGACGACCTGGTTACTCCTTGAG |
| ICAM-1 | AGTGACTGTCACTCGAGATCTTGAGGGC |
|  | GCCTGCAGTGCCCATTATGACTGCGG |
